# Hata-Yanagiya physical activity calculation system: a novel global positioning system-based method for accurate estimation of oxygen consumption during walking and running

**DOI:** 10.3389/fspor.2024.1522214

**Published:** 2025-01-10

**Authors:** Keiichiro Hata, Toshio Yanagiya, Hiroaki Noro, Yoshio Suzuki

**Affiliations:** ^1^ Graduate School of Health and Sports Science, Juntendo University, Inzai, Japan; ^2^ Institute of Health and Sports Science & Medicine, Juntendo University, Inzai, Japan; ^3^ Faculty of Health and Sports Science, Juntendo University, Inzai, Japan

**Keywords:** HYPAC system, oxygen consumption, GPS, energy expenditure, marathon, metabolic equivalents, METs, physical activity

## Abstract

**Introduction:**

Marathon running has become increasingly popular among amateur athletes, many of whom maintain speeds of 8–9 km/h. However, existing methods for estimating oxygen consumption (VO_2_) during running and walking—such as the American College of Sports Medicine (ACSM) equations and commercial activity monitors—often lack accuracy and transparency. This study introduces the Hata-Yanagiya Physical Activity Calculation (HYPAC) system, a novel approach for estimating VO_2_ using Global Positioning System (GPS) and map data.

**Methods:**

The HYPAC system was developed through regression equations based on metabolic equivalents (METs) and slope data. To validate the system, 10 university students (5 runners, 5 non-runners) completed a 5 km course while equipped with a GPS device and a portable metabolic measurement system. VO_2_ estimates from the HYPAC system were compared with measured values and those calculated using ACSM equations.

**Results:**

The HYPAC system demonstrated high accuracy in estimating VO_2_, with a relative error of −0.03 [95% confidence intervals (CI): −0.14, 0.08] compared to measured values. For the running group, the HYPAC system achieved the lowest absolute mean relative error (0.02). In the mixed running/walking group, the HYPAC system maintained strong performance with a relative error of −0.07 (95% CI: −0.26, 0.12).

**Discussion:**

The HYPAC system provides a transparent and accurate method for estimating VO_2_ during walking and running, outperforming existing methods under varied conditions. Its open-source framework encourages further validation and improvement by researchers and practitioners. Future studies should address limitations such as sample size and population diversity to enhance the system's applicability.

## 1 Introduction

Marathon running, a demanding endurance sport, has become increasingly popular worldwide, providing participants with immense satisfaction upon completing the 42.195-km course. Driven by rising health consciousness and the appeal of personal challenge, many amateur runners now undertake full marathons. Most amateur events set a time limit of approximately six hours, with a substantial portion of finishers maintaining an average pace of 8–9 km/h. For example, at the 17th Shonan International Marathon in Kanagawa, Japan, held on December 4, 2022, the average speeds for male and female runners were 9.4 ± 1.8 km/h (*n* = 10,585) and 8.6 ± 1.4 km/h (*n* = 1,645), respectively. Among these, 41.7% of men and 55.7% of women finished with an average speed between 7 and 9 km/h, according to the official event data (1).

The American College of Sports Medicine (ACSM) provides equations for estimating VO_2_ during walking and running (2):

***Walking (<8 km/h)***

$$\begin{array}{c} VO_{2}\;(\mathrm{ml}/\mathrm{kg}/\min)=\mathrm{Speed}\;(m/\min)\times 0.1\;(\mathrm{ml}/\mathrm{kg}/m)\\ +\;\mathrm{Speed}\;(m/\min)\times \mathrm{Slope}\;(\mathrm{decimal})\times 1.8\;(\mathrm{ml}/\min/m)+3.5\;(\mathrm{ml}/\mathrm{kg}/\min) \end{array}$$

***Running (≥8 km/h)***

$$\begin{array}{c} VO_{2}\;(\mathrm{ml}/\mathrm{kg}/\min)=\mathrm{Speed}\;(m/\min)\times 0.2\;(\mathrm{ml}/\mathrm{kg}/m)\\ +\;\mathrm{Speed}\;(m/\min)\times \mathrm{Slope}\;(\mathrm{decimal})\times 0.9\;(\mathrm{ml}/\min/m)+3.5\;(\mathrm{ml}/\mathrm{kg}/\min) \end{array}$$

However, an inconsistency arises at exactly 8 km/h with a flat slope (0), yielding VO_2_ values of 16.8 ml/kg/min for walking and 30.2 ml/kg/min for running, potentially causing errors around this speed. Various commercial activity monitors also estimate energy expenditure during exercise. Typically, worn on the wrist or arm, these devices utilize acceleration sensors, heart rate monitors, or measures like heat or galvanic skin response to estimate energy consumption. However, these devices tend to underestimate energy consumption, and discrepancies among devices are common (3). Additionally, none of these devices disclose their calculation algorithms.

In soccer, Global Positioning System (GPS)-based devices estimate physical activity based on movement data, though they often underestimate energy expenditure. Accuracy improves when anaerobic components, such as excess postexercise oxygen consumption, are excluded (4); however, these devices also lack published calculation algorithms. Consequently, no scientifically validated method currently exists to reliably measure VO_2_, and thereby energy expenditure, for recreational marathon runners.

A GPS device can record the latitude, longitude, and time of a marathon runner with high temporal resolution. Altitude data at each GPS-measured point can be derived from map information, allowing for the calculation of altitude differences and slope between points. Therefore, both speed and slope between each measured point can be determined from GPS and map data. The metabolic equivalents (METs) for various physical activities are cataloged in the Compendium of Physical Activities (5), an internationally recognized resource that includes METs for horizontal walking and running at different speeds. Additionally, VO_2_ requirements for walking and running on slopes at various speeds have been documented (6).

We hypothesized that a regression equation for estimating VO_2_ during walking and running could be developed using the METs table and slope data from Minetti et al. (6), enabling VO_2_ estimation based on speed and slope from GPS and map data. In this study, we present the Hata-Yanagiya Physical Activity Calculation (HYPAC) system, a calculation method derived from regression equations based on this hypothesis, and validate its effectiveness in a sample of university students.

## 2 Methods

### 2.1 HYPAC

**Figure 1:**
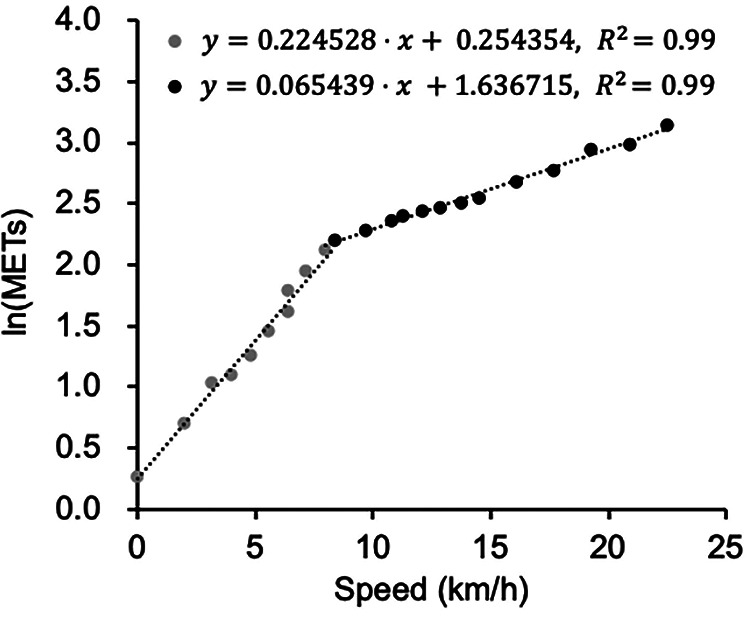
The relationship between speed and ln(METs). Gray dots represent speeds <8.69 km/h, while black dots indicate speeds ≥8.69 km/h.

The exercise intensities for various horizontal speeds and standing still were extracted from the Compendium of Physical Activities (Supplementary Table S1). Although no single equation accurately represented the data, two regression models using the natural logarithm of METs as the dependent variable (Equations 1, 2) demonstrated a strong fit, with high coefficients of determination, using 8.69 km/h as a threshold (Figure 1). VO_2_ was calculated with the standard conversion of 1 MET to 3.5 ml/kg/min.

***Speed < 8.69 km/h***

(1) $$\begin{array}{c} \ln\;(\mathrm{METs})=0.224528\times \mathrm{Speed}\;(\mathrm{km}/h)+0.254354\\ R^{2}=0.985 \end{array}$$

***Speed ≥ 8.69 km/h***

(2) $$\begin{array}{c} \ln\;(\mathrm{METs})=0.065439\times \mathrm{Speed}\;(\mathrm{km}/h)+1.636715\\ R^{2}=0.993 \end{array}$$

**Figure 2:**
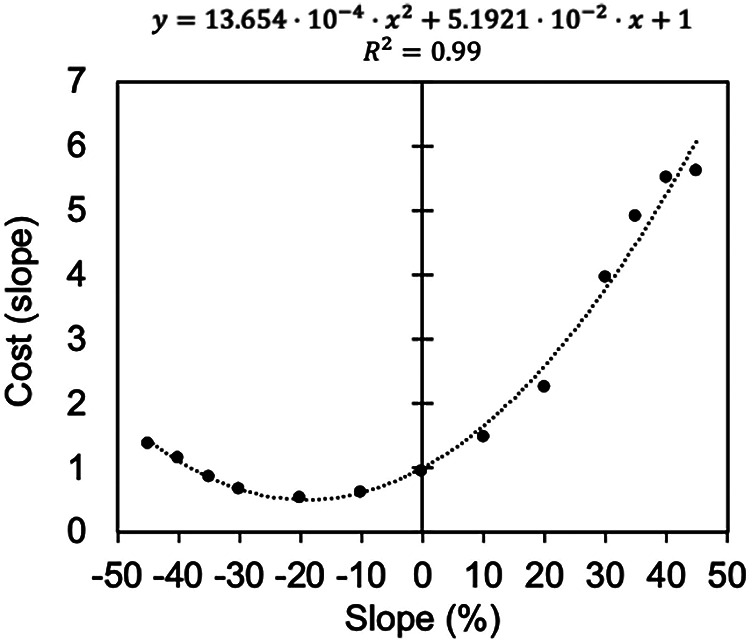
Relationship between slope and cost (slope) in terms of VO_2_. The horizontal axis represents the slope (%), and the vertical axis represents the slope cost in VO_2_ terms.

Minetti et al. reported VO_2_ values for running at various speeds on gradients up to ±45% (6). VO_2_ for horizontal travel at each speed was determined using regression equations (Equations 1, 2) and then compared with the VO_2_ values from Minetti et al. (6). The cost of slope, defined as the ratio of oxygen consumption for inclined travel relative to horizontal travel, was closely approximated by a quadratic regression curve with a Y-intercept of 1, using slope (%) as the independent variable (Equation 3; Figure 2):

(3) $$\begin{array}{c} \mathrm{Cost}\;(\mathrm{slope})=13.6524\times 10^{-4}\times \mathrm{Slop}e^{2}+5.1921\times \cdot 10^{-2}\times \mathrm{Slope}+1\\ R^{2}=0.99 \end{array}$$

where:

Slope is defined as (vertical/horizontal) × 100 (%).

Based on this relationship, exercise intensity during running or walking on a slope was calculated as follows (Equation 4):

(4) $$\mathrm{MET}s_{\mathrm{slope}}=\mathrm{MET}s_{\mathrm{horizon}}\times \mathrm{Cost}\;(\mathrm{slope})$$

where:

METs_slope_: METs on a slope

METs_horizon_: METs for horizontal movement calculated from speed (km/h) using Equations 1, 2

Cost (slope): The VO_2_ rate in slope travel compared to horizontal travel

Using these equations, a system was developed to calculate VO_2_ from GPS and map data. For each GPS-measured coordinate, altitude data can be obtained via the application program interface of the Geospatial Information Authority of Japan (7). From this data, the Euclidean distance, slope, and speed between each adjacent point were calculated. Exercise intensity (METs × h) for each segment can then be computed, and the total exercise (METs × h) for on-foot travel as measured by GPS can be obtained. VO_2_ for the entire travel can be calculated using the individual's body mass and a standard VO_2_ value of 3.5 ml/kg/min per 1 MET.

This novel calculation method, termed the HYPAC system, is available as a Python script on GitHub (https://github.com/KH-SPORTSBIOMECH/HYPAC-Hata-YanagiyaPhysicalActivityCalculationSystem; (8).

### 2.2 Validation of the HYPAC system

#### 2.2.1 Participants

Ten healthy university students participated in this study (Age: 23.7 ± 2.4 years, Height: 1.67 ± 0.10 m, Body Weight: 59.7 ± 7.0 kg). Their characteristics were summarized in Table 1. Five males were recreational runners with regular exercise habits (Participants A to E in Table 1), while one male and four females had no exercise habits (Participants F to J in Table 1). Body mass measurements included the apparatus, shoes, and gear. Percentage body fat was assessed using a body composition analyzer (InBody 730, InBody Japan Inc., Japan). All participants completed the study and were included in the analysis. The study was approved by the Ethics Committee of Juntendo University Graduate School of Health and Sport Science (approval code: 2023-143) and conducted in accordance with the Declaration of Helsinki.

**Table 1:** Characteristics of participants.

ID	Sex	Age (year)	Height (m)	Body weight (kg)	Body mass (kg)^a^	Percentage body fat (%)
A	Male	26	1.72	60.3	62.1	14.1
B	Male	23	1.68	64.1	66.2	13.3
C	Male	22	1.69	62.6	64.8	13.1
D	Male	22	1.79	66.8	69.3	15.6
E	Male	22	1.85	65.7	67.8	8.9
F	Female	26	1.53	59.8	61.3	33.1
G	Female	24	1.56	53.7	55.7	31.8
H	Female	29	1.58	54.9	57.0	23.8
I	Female	21	1.54	43.0	44.6	15.9
J	Male	22	1.73	65.9	67.7	13.7
Mean		23.7	1.67	59.7	61.7	18.3
SD		2.4	0.10	7.0	7.2	7.9

^a^ Body mass measurements included the apparatus, shoes, and gear.

#### 2.2.2 5-kilometer run and walk

Participants were divided into two groups: a running group (participants A to E) and a running/walking group (participants F to J). GPS data and VO_2_ measurements were collected using a wearable smartwatch device (Garmin Foreathlete 745, Garmin Ltd., USA) and a breath-by-breath wearable metabolic system K5 (Cosmed, Italy)—the golden standard system for oxygen consumption, respectively.

Participants traveled a 5-kilometer (km) route that included downhill, uphill, and flat terrain. The running group ran at a moderate speed, with the requirement of completing the route without walking, while the running/walking group completed the route by alternating between running and walking, according to individual preferences and abilities. The route was set up using a route within our university and an outdoor route with minimal car traffic. During the measurement, we followed the subject on a bicycle to ensure the safety of the participants and give directions.

#### 2.2.3 Data analysis

VO_2_ values were calculated by the HYPAC system using GPS data obtained from the 5 km runs and walks. For comparison, VO_2_ was also calculated using the ACSM method, which included two equations based on a speed threshold of 8 km/h. For each adjacent point, VO_2_ was calculated using the ACSM_RW_ equation, while the ACSM_Run_ equation was applied only to speeds of 8 km/h and above.

The VO_2_ calculated by each of the three methods (HYPAC, ACSM_RW_, and ACSM_Run_) was then compared with the measured VO_2_ obtained from the K5. The distribution of VO_2_ values for K5, HYPAC, ACSM_RW_, and ACSM_Run_ was assessed using the Shapiro–Wilk test, which indicated that normality could be assumed. Relative error against K5, calculated as (Method—K5)/K5, was tested with a one-sample *t*-test, using 0 as the reference value. A generalized linear model was applied to compare the VO_2_ values obtained using the four methods and to analyze differences between each method and the K5 measurements. These statistical analyses were conducted using IBM SPSS version 29.0 (IBM Japan, Tokyo, Japan), with significance set at less than 0.05. Bland–Altman plot was used to analyze the agreement in VO_2_ between each method and K5 measurement using MATLAB (MATLAB R2021b, MathWorks, USA).

## 3 Results

The VO_2_, mean speed, and finish time for each participant are presented in Table 2.

**Table 2:** The VO_2_, mean speed, and finish time for each participant.

ID	VO_2_ (ml/kg/min)				Mean speed (km/h)
	K5	HYPAC	ACSM_RUN_	ACSM_RW_	
A	1,214.9	1,099.6	1,143.3	1,135.7	11.4
B	1,173.4	1,026.5	1,106.6	1,099.0	11.2
C	1,102.0	1,058.8	1,123.8	1,123.2	10.9
D	975.0	1,052.1	1,155.8	1,152.8	14.6
E	842.9	1,071.6	1,024.1	1,021.5	17.4
F	1,280.8	1,047.3	1,189.9	914.4	7.2
G	1,275.4	1,020.7	1,172.1	780.6	5.8
H	893.2	990.4	1,044.6	939.8	9.0
I	1,067.5	1,152.1	1,267.7	1,106.7	7.7
J	1,285.2	1,079.4	1,161.4	877.7	6.3
Mean	1,111.0	1,059.8	1,138.9	1,015.1	10.2
SD	155.1	42.8	66.7	122.6	3.6

**Figure 3:**
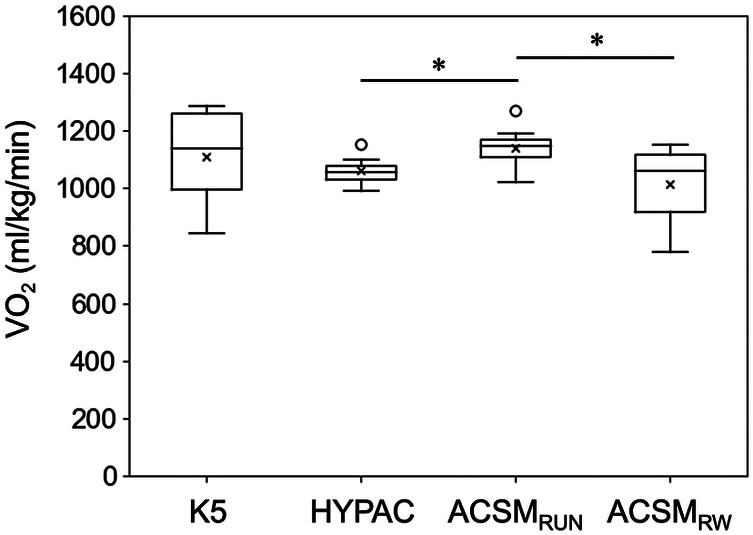
VO_2_ measured by K5 and calculated by HYPAC, ACSM_RW_, and ACSM_Run_. *Indicates significant differences between methods.

The VO_2_ measured by K5 showed no significant differences from the VO_2_ values calculated by HYPAC, ACSM_RW_, or ACSM_Run_. However, ACSM_Run_ values were significantly higher than those calculated by HYPAC (*p* < 0.001) and ACSM_RW_ (*p* = 0.029) (Figure 3).

**Figure 4:**
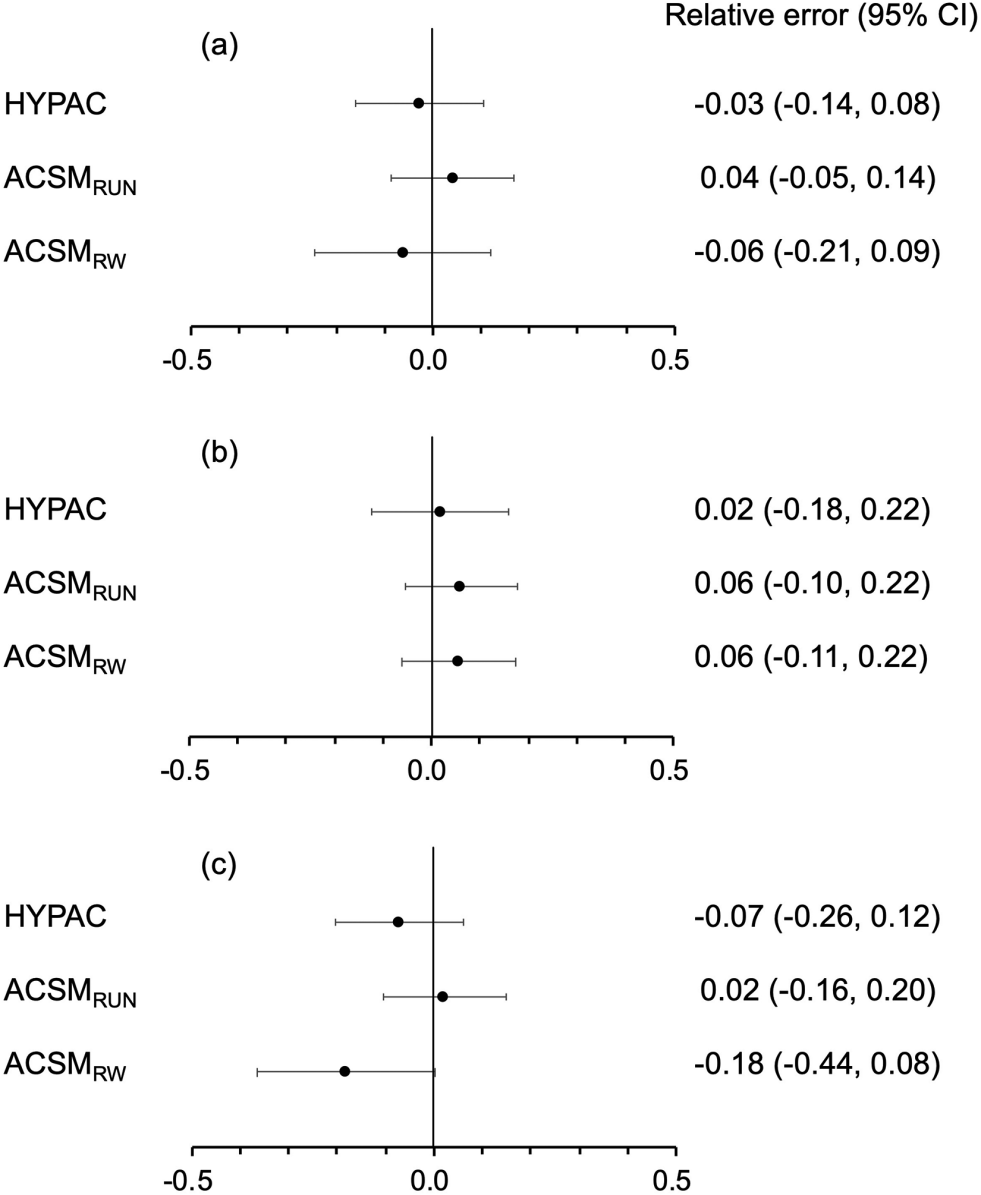
Relative errors and 95% confidence intervals (CI) in VO_2_ for HYPAC, ACSM_Run_, and ACSM_RW_ compared to K5: **(a)** whole group, **(b)** running group, and **(c)** running/walking group.

Relative errors and 95% confidence intervals (CIs) for HYPAC, ACSM_Run_, and ACSM_RW_ in VO_2_ compared to K5 were shown in Figure 4.

Across all groups, including both the running and running/walking groups, HYPAC had the smallest mean relative error (0.03) against K5, while ACSM_RW_ showed the largest (0.06) (Figure 4a). When analyzed separately, the running group (mean speed: 10.9–17.4 km/h) had a smaller mean relative error with HYPAC (0.02) than with ACSM_Run_ and ACSM_RW_ (Figure 4b). Notably, in the running/walking group (mean speed: 5.8–9.0 km/h), ASCM_Run_ showed the smallest error (0.02), while ACSM_RW_ had the largest error (0.18) (Figure 4c).

Statistical analysis showed no significant differences in the relative error against K5 for any of the methods, nor were there significant differences between the methods in terms of relative error against K5.

**Figure 5:**
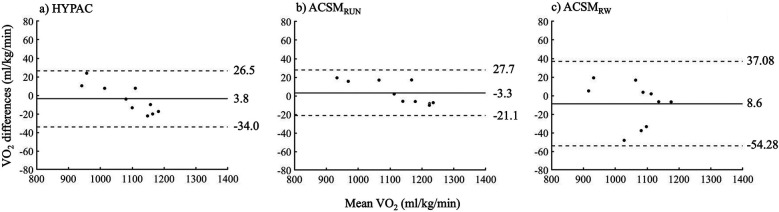
Bland–Altman plot of differences in VO_2_ between the K5 and HYPAC **(a)**, ACSM_RUN_ **(b)**, ACSM_RW_ **(c)** the solid line indicates the bias, which refers to the systematic difference (mean difference) between the K5 method and each method for calculating VO_2_. The dashed lines indicate the limits of agreement (±1.96 standard deviation).

From the Bland–Altman analysis, all samples for each method for VO_2_ estimation were distributed within the limits of agreement, confirming a strong agreement in VO_2_ between the K5 and each method (Figure 5).

## 4 Discussion

In this study, the HYPAC system demonstrated a low relative error in estimating VO_2_ during both walking and running compared to the K5 respiratory gas meter, particularly during running. Energy expenditure, calculated from VO_2_ using a conversion rate of 5 kcal/L of oxygen consumed, can therefore be accurately estimated with the HYPAC system during these activities.

As mentioned in the Introduction, the ACSM formula has long been the standard for estimating VO_2_ in walking and running (2), providing two equations—one for walking (<8 km/h) and one for running (≥8 km/h). However, a notable gap exists at 8 km/h on level ground, where the calculated VO_2_ jumps from 16.8 ml/kg/min (walking) to 30.2 ml/kg/min (running) (Supplementary Table S1). Given that the average speed for many amateur marathon runners is between 7 and 9 km/h, the applicability of the ACSM formula to all marathon runners is unclear. For instance, at the 2022 Shonan International Marathon, this speed range was common (1). Similarly, approximately 11% of the 26,622 finishers in the 2023 Boston Marathon ran between 7 and 9 km/h (9). Therefore, the ACSM formula may not accurately estimate VO_2_ of all marathon runners.

The HYPAC system aims to provide accurate VO_2_ estimates for walking and running by calculating METs for horizontal movement based on speed and adjusting for incline. In this study, participants walked and ran on a 5 km road course at average speeds ranging from 5.8 to 17.4 km/h, and their VO_2_ measurements from the HYPAC system closely aligned with K5 measurements, showing a relative error of only 3%.

While numerous wearable devices are available to measure physical activity, they often show significant variability. The accuracy of wrist-worn GPS devices is high and continues to improve. According to a 2013 report, the relative error of the Garmin® Forerunner 110 ranged from 0.8% to 6.2% (10). A 2020 study comparing different models reported that the relative error of GPS in wearable devices ranged from 0.6% ± 0.3% to 1.9% ± 1.5% (11). Regarding energy expenditure, a 2020 meta-analysis by O'Driscoll et al. reported a pooled mean bias, Hedges’ g, for running of −0.08. Although the differences from the reference measurements (indirect calorimetry, room calorimeters, and doubly labeled water) were minor, significant heterogeneity was observed across devices (3). Additionally, the 95% CIs for each device indicated a standard error greater than 0.3 for all devices (3). In a recent review by Germini et al., a meta-analysis could not be performed due to large heterogeneity between devices, and the mean absolute percentage error exceeded 30% for all devices in estimating energy consumption (12). In addition to this substantial margin of error, the calculation methods used in commercially available devices have not been disclosed.

In contrast, the HYPAC system demonstrated a higher degree of accuracy in estimating VO_2_, with a relative error of −0.03 (95% CI: −0.14, 0.08), achieved by directly measuring VO_2_ rather than relying on indirect methods. The Python code for the HYPAC system used in this study is also available on GitHub (https://github.com/KH-SPORTSBIOMECH/HYPAC-Hata-YanagiyaPhysicalActivityCalculationSystem) (8). This allows researchers and practitioners to verify and enhance the system. Therefore, the HYPAC system is superior to commercially available devices for estimating VO_2_ during walking and running, as its calculation methods are transparent and validated.

Furthermore, when walking and running were mixed, the estimate using ACSM_Run_ was closest to the actual VO_2_. Since ACSM_Run_ is a formula applicable to running speeds of 8 km/h or more, VO_2_ was likely overestimated when walking was included at speeds below 8 km/h. We hypothesized that ACSM_RW_, a modification of the ACSM formula based on speed, would provide more accurate estimates; however, this hypothesis was rejected. Although the HYPAC system had lower estimation accuracy than ACSM_Run_ in this mixed walking/running condition, the relative error in actual VO_2_ was −0.07 (95% CI: −0.26, −0.12), which is considered sufficiently acceptable. Additionally, the HYPAC system, like ACSM_Run_, underestimated VO_2_. Therefore, the HYPAC system may require further refinement to accommodate variations in speed and activity. However, these results were derived from a small sample size (*n* = 5). Further research is warranted to re-examine the applicability of ACSM_Run_ at speeds of 8 km/h or greater in mixed walking and running conditions with a larger sample size. In addition, The Bland–Altman analysis confirmed a strong agreement in VO₂ between the K5 and each method. The bias in VO₂ measurements was 3.8 ml/kg/min for the HYPAC method and −3.3 ml/kg/min for the ACSM_RUN_ method, both of which represent small biases. A trend was observed where both the HYPAC and ACSM_RUN_ methods tended to overestimate below approximately 1,100 ml/kg/min and underestimate them above approximately 1,100 ml/kg/min compared to the values measured by K5. However, the small sample size may have contributed to this trend.

When comparing VO_2_, the normality of the distribution could be assumed, but this may be due to the small sample size. Therefore, we compared the methods using the distribution-free generalized linear model. Several factors, such as weight, sex, and body composition, could confound the comparison. However, due to the small sample size, which may lead to overfitting, these factors were not controlled for in the comparison between methods using the generalized linear model. Instead, all data were presented in tabular form, allowing researchers to make their own judgments.

This study has several limitations. First, the sample size for the validity study was small, with *n* = 5 for each of the running and walking/running groups. This may have resulted in wider 95% CIs for relative error. The sample size needs to be increased to provide more accurate measurement accuracy. Second, the participants in this study were physically active university students with a narrow age range. Therefore, further research is needed to adapt the HYPAC system to other subjects, such as older recreational runners and sedentary individuals. In addition, this study did not examine the effects of sex or body composition. The METs that form the basis of the HYPAC system are defined based on VO_2_ at rest and are not affected by sex. Although body fat consumes less oxygen than lean mass, it contributes to total VO_2_ as weight. The GPS data was collected using Germin, but there may be a potential for bias in GPS data. Therefore, in order to clarify and further improve the characteristics of the HYPAC system, it is necessary to also consider the effects of sex differences and body fat percentage.

## 5 Conclusion

This study demonstrated that the HYPAC system, which uses estimation formulas for VO_2_ based on published VO_2_ data during exercise, along with speed and elevation data obtained from GPS-map data at nearly one-second intervals, can estimate VO_2_ during running and walking with high accuracy, showing a relative error of −0.03 (95% CI: −0.14, 0.08). The HYPAC system offers a transparent, evidence-based calculation method, and the Python script has also been made publicly available. However, there remains significant limitations due to small sample size and homogeneity of the participants. Consequently, further dissemination, verification, and refinement of the system are expected in the future.

## Data Availability

The original contributions presented in the study are included in the article/Supplementary Material, further inquiries can be directed to the corresponding author.
