# Supplementary material for: Hata-Yanagiya physical activity calculation system: a novel global positioning system-based method for accurate estimation of oxygen consumption during walking and running
Source: Front Sports Act Living. 2025 Jan 10;6:1522214. doi: 10.3389/fspor.2024.1522214 (PMC11757879; doi:10.3389/fspor.2024.1522214)
Supplement: Supplementary file 1 [file Table1.docx]

**Supplementary Figure 1**. The gap in oxygen consumption (VO_2_) measurements between the two ACSM formulas.

In this linear regression, the red line represents the ACSM walking formula (< 8 km/h), while the black line indicates the ACSM running formula (≥ 8 km/h) for estimating VO_2_. A gap in VO_2_ measurements appears at approximately 6 to 8 km/h, which corresponds to the average speed of most marathon participants. VO_2_: oxygen consumption; ACSM: American College of Sports Medicine
