# Supplementary material for: Hata-Yanagiya physical activity calculation system: a novel global positioning system-based method for accurate estimation of oxygen consumption during walking and running
Source: Front Sports Act Living. 2025 Jan 10;6:1522214. doi: 10.3389/fspor.2024.1522214 (PMC11757879; doi:10.3389/fspor.2024.1522214)
Supplement: Supplementary file 2 [file Table2.docx]

# Supplementary table 1. Extracted run/walk METs and speed from the Compendium of Physical Activity

| CODE | METs | Activity | Speed（km/h） |
| --- | --- | --- | --- |
| 7040 | 1.3 | standing quietly | 0.0 |
| 17151 | 2.0 | Walking | 2.4 |
| 17152 | 2.8 | Walking | 3.2 |
| 17170 | 3.0 | Walking | 4.0 |
| 17190 | 3.5 | Walking | 4.8 |
| 17200 | 4.3 | Walking | 5.6 |
| 12029 | 6.0 | Running | 6.4 |
| 17220 | 5.0 | Walking | 6.4 |
| 17230 | 7.0 | Walking | 7.2 |
| 17231 | 8.3 | Walking | 8.0 |
| 12030 | 8.3 | Running | 8.0 |
| 12040 | 9.0 | Running | 8.4 |
| 12050 | 9.8 | Running | 9.7 |
| 12060 | 10.5 | Running | 10.8 |
| 12070 | 11.0 | Running | 11.3 |
| 12080 | 11.5 | Running | 12.1 |
| 12090 | 11.8 | Running | 12.9 |
| 12100 | 12.3 | Running | 13.8 |
| 12110 | 12.8 | Running | 14.5 |
| 12120 | 14.5 | Running | 16.1 |
| 12130 | 16.0 | Running | 17.7 |
| 12132 | 19.0 | Running | 19.3 |
| 12134 | 19.8 | Running | 20.9 |
| 12135 | 23.0 | Running | 22.5 |
